# Optimal Foraging Predicts the Ecology but Not the Evolution of Host Specialization in Bacteriophages

**DOI:** 10.1371/journal.pone.0001946

**Published:** 2008-04-16

**Authors:** Sébastien Guyader, Christina L. Burch

**Affiliations:** Department of Biology, University of North Carolina, Chapel Hill, North Carolina, United States of America; University of Ottawa, Canada

## Abstract

We explore the ability of optimal foraging theory to explain the observation among marine bacteriophages that host range appears to be negatively correlated with host abundance in the local marine environment. We modified Charnov's classic diet composition model to describe the ecological dynamics of the related generalist and specialist bacteriophages φX174 and G4, and confirmed that specialist phages are ecologically favored only at high host densities. Our modified model accurately predicted the ecological dynamics of phage populations in laboratory microcosms, but had only limited success predicting evolutionary dynamics. We monitored evolution of attachment rate, the phenotype that governs diet breadth, in phage populations adapting to both low and high host density microcosms. Although generalist φX174 populations evolved even broader diets at low host density, they did not show a tendency to evolve the predicted specialist foraging strategy at high host density. Similarly, specialist G4 populations were unable to evolve the predicted generalist foraging strategy at low host density. These results demonstrate that optimal foraging models developed to explain the behaviorally determined diets of predators may have only limited success predicting the genetically determined diets of bacteriophage, and that optimal foraging probably plays a smaller role than genetic constraints in the evolution of host specialization in bacteriophages.

## Introduction

The advantages of host range expansion for parasites include reduced intraspecific competition for hosts, reduced time spent searching for hosts, and an increased probability of transmission to a suitable host [1]. Despite these advantages, parasites are most often observed to specialize on a limited number of host species. Explanations for the prevalence of host specialists may be ecological or evolutionary, and include ecological tradeoffs between diet breadth and diet quality [2], and genetic tradeoffs in the ability to utilize alternative hosts [3], [4]. Unlike investigations of predator foraging strategies that focus on the ecological explanations for specialization, investigations of parasite foraging strategies have focused on evolutionary explanations [e.g. 3], [4] often ignoring ecological explanations entirely [but see 5], [6]. The reason for this difference in focus is most likely methodological. Parasites are more amenable to genetic analysis and manipulation than predators, but parasites are less amenable to ecological observation.

The ecological explanation for the prevalence of host specialists comes from an optimal foraging model developed to describe the consequences of predator diet compositions. The model explores the trade-off between diet breadth and diet quality, and generally predicts that predators should specialize on a small number of highly profitable prey types unless prey abundance is low. Diet composition models have accurately predicted the diet composition of predators such as bluegill sunfish [7], [8] and great tits [9], and should be equally applicable to parasites [e.g. 6], [e.g. 10] as long as their encounter with hosts is governed by a first-order process in which encounter rates increase linearly with host abundance [11]. Thus, diet composition models should accurately describe the ecology of parasites that are transmitted through the environment, but may not apply to parasites that require direct contact between infected and uninfected hosts or transmission by a specialist insect vector.

In this study we investigated the ability of optimal foraging theory to explain the prevalence of host specialists among bacteriophage, parasites that are both transmitted through the environment and amenable to experimental manipulation. Our interest in bacteriophages arose from recent observations that phages collected from higher host density coastal waters have a greater tendency toward host specialization than phages collected from lower host density pelagic ocean waters [12]–[14]. The major observations come from studies that sampled phages by performing plaque assays on numerous bacterial strains that were co-isolated from the same marine waters, as in [15]. The isolated phages were then tested for the ability to grow on bacterial strains other than the strain used to isolate that phage. Only 41 out of 217 phages (18.9%) collected from low nutrient pelagic waters of the North Atlantic were specialists that grew only on the original host strain [16], whereas 88 out of 194 (45%) phages collected from productive waters off the German coast were host specialists [12], [17]. These data suggest a relationship between host density and host specialization, as long as bacterial diversity was not higher in the low density environment – a possibility that seems unlikely [18], [19].

Although optimal foraging theory offers an intuitively appealing explanation for this observed relationship between host density and host specialization, even the richest marine environments achieve host densities that seem too low to explain the prevalence of specialist phages in the marine environment [14]. This latter observation suggests that either the ecological dynamics of bacteriophage populations are not accurately described by diet composition models, or that the evolutionary dynamics are not well predicted by such models. To distinguish between these possibilities, we tested the predictions of a diet composition model by monitoring the ecological and evolutionary dynamics in laboratory microcosms of populations of two related phages, the generalist phage φX174 that infects both *Escherichia coli* and *Salmonella enterica* serovar Typhimurium (hereafter *S. typhimurium*), and the specialist phage G4 that infects only *E. coli*.

### Optimal foraging in bacteriophages

In order to use the diet composition model of Charnov [11] to describe the consequences of host specialization for bacteriophages, we modified the model slightly to account for the exponential growth dynamics of phages. Although the qualitative prediction that host specialization should be positively correlated with host abundance remains unchanged, the modification is necessary to quantify the host density above which specialization is favored. In Charnov's model, diet breadth changes as a function of the differences in search time (*s*), handling time (*h*), and energy content (*E*) between alternative hosts. These parameters are directly related to the phage life history parameters attachment rate (*k*), lysis time (*L*), and burst size (*B*), respectively.

In phages, attachment to a host bacterium is governed by first-order kinetics [20], thus phages bind to bacterial hosts according to the equation:
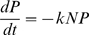
(1)where *P* is the density of unattached phage, *N* is the density of hosts, and *k* is an attachment rate constant. Search time can be calculated from equation 1 as the average time required for attachment to a host:
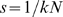
(2)Following attachment to a host, phages replicate inside the host until they lyse (kill) the host cell. Thus, handling time is equivalent to lysis time (*L*), the time that elapses from attachment to lysis. Finally, the burst size (*B*), or number of progeny released after lysis is equivalent to the relative energy content of the host.

Foraging in phages differs somewhat from the original diet width models [21], [22] because phage growth rates do not increase linearly with the energy content of the host. Instead, within-host replication of phages leads to an exponential increase in progeny numbers. In this case, phages increase over time according to:
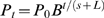
(3)where *P*_0_ and *P_t_* are the number of phages present initially and at time *t*. Note that *t*/(*s*+*L*) is the average number of generations (*g*) achieved in *t* minutes, and that the number of progeny produced by a single phage in *t* minutes is *B^g^*. Because phage populations grow exponentially, the rate of energy intake can be linearized by considering the rate of change in ln(*P*):
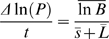
(4)where 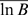 is the average energy content of hosts included in the current diet, and 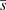 and 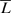 are the average search and handling times for hosts already included in the diet.

Using this framework we now consider the following question: Given that the *i*−1 most profitable hosts – ranked in decreasing order according to their profitability = ln(*B_i_*)/*L_i_* – are already included in the diet, should the phage expand its diet by including the next most profitable host as well? To answer this question, imagine that the phage has encountered (attached to) a new host of rank *i*. If the phage consumes this host, it will achieve an instantaneous rate of energy intake of ln(*B_i_*)/*L_i_*. If the phage, instead, ignores this host and continues to search for a more profitable host, it will spend 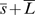 time searching for and handling a more profitable host in order to take in 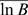 energy content. In this scenario the optimal strategy is to consume hosts of rank *i* if, and only if,
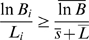
(5)


The primary prediction of equation 5 is that host range should be negatively correlated with host abundance. When host abundance is low, search times are large, making it easy to satisfy the inequality. Bacteriophage should consume any host they encounter. As host abundance increases, search times approach zero, and it becomes increasingly difficult to satisfy the inequality. In this case, bacteriophage should consume only the most profitable hosts. We note that these are the classical predictions of the diet composition model [21]. We have only fitted the classical model to describe the particular case of bacteriophage dynamics.

In the experiments that follow, we use equation 5 to identify the threshold host density above which specialists should be favored in laboratory microcosms containing two hosts. We then test the primary prediction of the model – that host range should be negatively correlated with host abundance – by examining the ecological and evolutionary dynamics of phage populations in laboratory microcosms maintained at host densities above and below this threshold.

## Methods

### Phage and bacterial strains

The bacteriophages φX174 and G4 are laboratory clones described in Bull et al. [23] and Holder and Bull [24], respectively. These phages and their standard laboratory hosts *E. coli* strain C and *S. typhimurium* strain LT2 were obtained from Holly Wichman (University of Idaho). φX174 infects both hosts; G4 infects only *E. coli* C.

### General culture conditions

All phages and bacteria were grown at 37°C with shaking in LB broth (10 g NaCl, 10 g Bacto-tryptone, and 5 g yeast extract per liter of water) supplemented with 2 mM CaCl_2_, and were stored in LB at 4°C for immediate use or in 4∶6 glycerol/LB (v/v) at −80°C for later use. The phage lysates used to initiate experiments were prepared by mixing approximately 10^3^ phages, 200 µL of an overnight *E. coli* culture (about 10^9^ bacteria/mL), and 3 mL of LB top agar (0.7% agar), and overlaying this mixture on an LB agar plate (1.5% agar). After a 4 hour incubation at 37°C, the top agar surface was harvested and mixed with 2 mL of LB. The top agar and bacteria were pelleted by centrifugation at 3000 rpm for 10 minutes, and the supernatant was stored as described. When needed, phage lysates were titered by plating a small amount of phages as described above, and counting the resulting number of plaques.

### Attachment Rate Assays

Attachment rate constants were determined as in [20]. Approximately 10^3^ phages were mixed with 1 mL of an exponentially growing culture at a density of ∼10^8^ bacteria/mL and incubated with shaking at 37°C. Immediately (time *t* = 0), and then again after a few minutes (2, 4, 6 and 8 minutes for foraging model parameterization; 5 minutes only for evolution experiments), 150 µL of this mixture was centrifuged for one minute at 10K rpm and 100 µL of the supernatant was plated to count the unbound phages. The rate at which the concentration of unbound phages decreases is determined by the following equation:
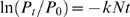
(6)where *P_t_* is the concentration of unbound phages at time *t* minutes, *k* is the attachment rate constant, and *N* is the concentration of bacteria. Therefore, we calculated the attachment rate constant (*k*) as the slope of the regression of ln(*P_t_*/*P_0_*) over time divided by the bacteria concentration, *N*. For each assay, *N* was measured by plating a 10^−6^ dilution of the bacterial cultures on LB plates. Attachment rates were measured in triplicate, except for the evolution experiment where it was measured one time from each of 6 independent plaque-purified clones.

### Lysis Time and Burst Size

**Figure 1:**
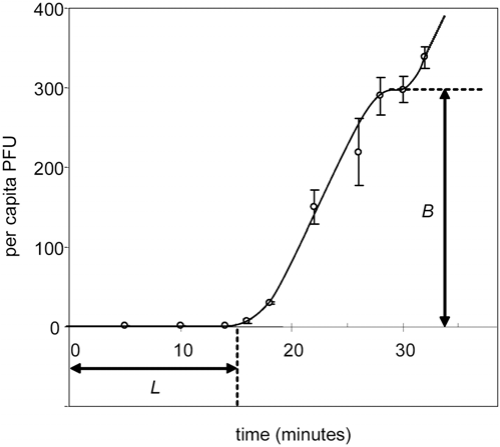
G4 growth curve illustrates how lysis time (*L*) and burst size (*B*) were determined. Data are means and standard errors for the number of plaque-forming units (PFU = number of infected cells+free phage particles) at various times during the course of infection. The curve was drawn by hand through the data for illustrative purposes. Two bursts occur during this time window. The first burst started around 15 minutes, the second burst started around 30 minutes.

Lysis times were estimated from one step growth curves (Figure 1). Approximately 10^3^ phages were mixed with 3 mL of an exponentially growing culture at a density of 10^8^ bacteria/mL, and incubated shaking at 37°C. Aliquots of this mixture were plated at different time intervals to monitor the increase in phage concentration. Preliminary experiments indicated that lysis time occurred at about 15 minutes, so we plated to estimate phage concentration every 5 minutes for the first 10 minutes (well before lysis), and every 2 minutes between 14 and 22 minutes, and between 26 and 32 minutes (the time intervals in which lysis events were expected to occur). An initial latent period in which the concentration of plaque forming units (PFU) remains constant was followed by a lysis period in which the PFU concentration increases sharply (note that before lysis, the number of progeny phages increases, but as they remain inside the same bacterial cell, the apparent count in PFU is unchanged). The first time point at which the phage concentration increased to more than twice the initial concentration was identified, and lysis time (*L*) was calculated as the mean between this time point (immediately post lysis) and the previous time point (immediately pre lysis). Burst sizes were also estimated from one step growth curves (Figure 1). Once all infected cells have been lysed, the concentration of PFU levels off and achieves a maximum value before the next lysis period begins. Burst size was then determined by dividing this maximum value (obtained at time *t* = 2*L*) by the initial PFU concentration. Lysis times and burst sizes were measured in triplicate.

### Growth Rate Assays

Host bacteria were grown to a density of 4×10^8^ bacteria/mL (exponential growth phase), at which point the appropriate volume of bacterial culture(s) was mixed with approximately 10^3^ phages (5×10^2^ φX174 and 5×10^2^ G4) and sterile LB, both preheated to 37°C, to achieve a final volume of 2 mL. The final bacterial density differed according to 4 different treatments (2 host densities×2 host compositions): 10^8^ *E. coli*/mL (high density×one host), 10^6^ *E. coli*/mL (low density×one host), 10^8^ *E. coli* and 10^8^ *S. typhimurium*/mL (high density×two hosts), or 10^6^ *E. coli* and 10^6^ *S. typhimurium*/mL (low density×two hosts). These mixtures were incubated shaking for 45 minutes at 37°C, allowing 2 full cycles of phage replication and release. The infection process was stopped by adding 50 µL of chloroform to each tube and vortexing. This process destroys bacterial cells, but does not release phage particles trapped inside infected cells. Initially (*t*_0_) and after 45 minutes (*t*_45_), phage concentrations were measured by plating on a mixed lawn consisting of 200 microliters of an overnight *E. coli* culture and 60 microliters of an onvernight *S. typhimurium* culture. 200 µL of *E. coli* ensures that both phages produce clearly visible plaques on the mixed lawn, and 60 µL of *S. typhimurium* is enough to make G4 plaques appear turbid, but not so much that G4 plaques are no longer clearly visible. φX174 forms clear plaques on this mixed lawn. The growth rates for each phage were calculated as the ratio of phage concentration at *t*_45_ to that at *t*_0_, and log transformed for statistical analyses. Each of these experiments were replicated in 5 complete blocks (*i.e.* one replicate of each treatment), with each block performed on a different day.

We measured the growth rates of φX174 and G4 simultaneously in individual assays to standardize the conditions experienced by generalist and specialist phages, but recognized that the presence of interspecific phages in each assay had the potential to reduce phage growth rates by increasing competition for hosts. To minimize this potential, we conducted the assays at a low multiplicity of infection (MOI, or ratio of phage to hosts) of 10^−5^. In addition, we confirmed that these mixed-phage assays yield the same results as assays conducted separately on the two phages by repeating a subset of the experiments using separate assays to measure the growth rates of φX174 and G4 (data not shown).

### Experimental evolution

Because the phages used in this study have a low spontaneous mutation rate and are genetically homogeneous (clones), we used chemical mutagenesis to generate genetic variation upon which selection could act. Virus lysates were treated with a 0.5 M hydroxylamine (HA) solution for 30 minutes, at which time 99% of phages were unable to form plaques. At this dose, phages receive an average of 4.65 lethal mutations per genome, and the surviving phages are expected to carry numerous non-lethal mutations. Even at an average dose of 1 lethal mutation per genome, 7% of the surviving phages acquire temperature sensitive mutations [25]. About 10^3^ mutagenized phages were plated and harvested in order to both eliminate remaining HA and obtain high titer lysates for experiments. Four independently mutagenized phage populations were obtained and used for serial passage experiments. High host density passages were initiated by adding 10^4^ mutagenized phages to 1 mL of LC containing 10^8^ cells each of *E. coli* and *S. typhimurium* (10^8^ bacteria/mL for each host type). Low host density passages were initiated by adding 10^4^ mutagenized phages to 100 mL containing 10^6^ cells each of *E. coli* and *S. typhimurium* (10^6^ bacteria/mL for each host type). We aimed to transfer 10^4^ phages after each generation (every 30 minutes) by diluting a small volume into new flasks containing host bacteria, so as to keep the MOI below 1 (actual MOI varied from 1.13×10^−4^ to 1.2×10^−1^). Ten passages were completed for each of the 4 replicate mutagenized populations, for each host density treatment. After completion of these experiments, samples were treated with chloroform and stored in 40% v/v glycerol/LC at −80°C.

We screened mutagenized phages for the ability to infect *S. typhimurium* by plating on mixed lawns of *E. coli* and *S. typhimurium*, and looking for clear plaques. Mutagenized phages must be screened on mixed lawns because they do not express their mutant phenotypes until after they replicate once in *E. coli*.

### Statistical analyses

All statistical tests were performed using SAS version 9.1 (SAS Institute, Cary, NC). ANOVAs were implemented using the GLM procedure, and the ANOVA assumptions of homogeneous and normally distributed residuals were confirmed using Shapiro-Wilk's and Levene's tests, respectively. In all cases, block was treated as a random factor and the other factors as fixed. Planned comparisons were made using the TDIFF and PDIFF statements to conduct t-tests and paired t-tests, respectively.

## Results

### Optimal foraging model predictions

To parameterize the optimal foraging model described above, we conducted attachment assays and single-step growth curves to measure attachment rates (*k*), lysis times (*L*), and burst sizes (*B*) of both phage (φX174 and G4) on both hosts (*E. coli* and *S. typhimurium*). The resulting parameter values are shown in Table 1. The measurements reveal two major properties of phage growth dynamics on these hosts. First, *E. coli* is a more profitable host than *S. typhimurium* for the generalist phage φX174. The higher profitability results both from a shorter handling time (*L*), and from a higher energy content (ln*B*) on *E. coli*. Second, G4 achieves a higher growth rate than φX174 on the shared host, *E. coli*. The higher growth rate of G4 results from a larger burst size; the two phage have similar attachment rates and lysis times on *E. coli*.

**Table 1:** Life history parameters of φX174 and G4.

Parameter	φX174		G4	
	*E. coli*	*S. typhimurium*	*E. coli*	*S. typhimurium*
*k* (×10^−9^ mL/min)	3.29±.581	2.14±.733	3.23±.746	-
*L* (min)	15	20	15	-
*B* (PFU)	170.06±7.76	131.73±1.62	297.77±16.76	-

All values are means of 3 replicates±SEM. Lysis times showed no detectable variation.

**Figure 2:**
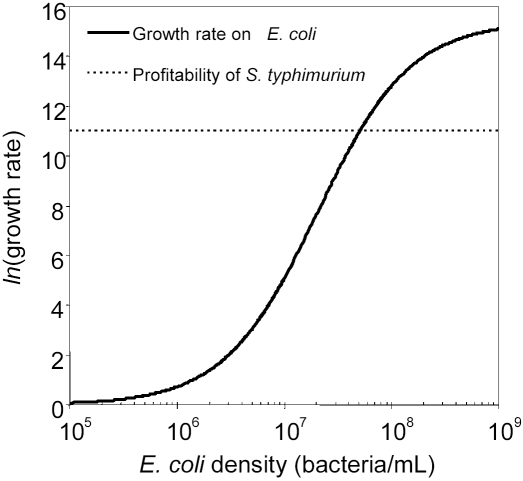
Predictions of the optimal foraging model using parameters measured in φX174. Given that φX174 consumes the more profitable host *E. coli*, we determined the *E. coli* densities over which φX174 benefits by also consuming the less profitable host *S. typhimurium*. φX174 should consume *S. typhimurium* only at host densities where the profitability of *S. typhimurium* (dashed line) exceeds the growth rate achieved on a diet of only *E. coli* (solid line). Both quantities are shown on the same scale as the experimental measures, in which growth rate is calculated as the phage concentration after 45 minutes divided by the initial phage concentration. Growth rate on *E. coli* was calculated as ln*B_Ec_*/(1/(*k_Ec_N*)+*L_Ec_*)×45 minutes, and profitability of *S. typhimurium* was calcualated as ln*B_St_*/*L_St_*×45 minutes. Both quantities are described in equation 5.

Incorporating the measured parameter values into the left and right sides of equation 5, we determined the density of *E. coli* below which it should be advantageous for φX174 to include *S. typhimurium* (the less profitable host) in the diet. In Figure 2 we plot the expected rate of energy intake as a function of *E. coli* density for a diet consisting only of *E. coli* (right side of equation 5). In addition we plot the profitability of *S. typhimurium* (left side of equation 5), a quantity that does not depend on host density. The two lines intersect at a host density of 5.03×10^7^ bacteria/mL, indicating that inclusion of *S. typhimurium* in the diet should be advantageous for *E. coli* densities below 5×10^7^ bacteria/mL. In other words, the optimal foraging model predicts that the generalist phenotype of φX174 will be advantageous below this cell density, but costly above it.

### Ecological dynamics

We measured the growth rates of the generalist (φX174) and specialist (G4) phages under conditions of high (10^8^ bacteria/mL) and low (10^6^ bacteria/mL) host densities, and in the presence or absence of *S. typhimurium*. We expect the presence of *S. typhimurium* to have a positive effect on phage growth rate at low host density and a negative effect at high host density (a host composition×host density interaction), but only for the generalist phage φX174. The specialist phage G4 provides a negative control in this experiment because it is expected to be unaffected by the presence of *S. typhimurium*. ANOVAs performed separately on the data collected for each phage confirmed these predictions (Table 2), revealing a significant host composition×host density interaction effect on the growth rate of φX174, but not on the growth rate of G4.

**Table 2:** ANOVA test of host density and diet effects on phage growth rates.

Phage	Source	d.f.	Mean Square	*F* value	*P*>*F*
φX174	Host Density	1	98.6568	1534.86	<0.0001
	Diet	1	0.0005	0.01	0.9312
	Block	4	0.1391	2.16	0.1352
	Density×Diet	1	0.8000	12.45	0.0042
	Error	12	0.0643		
G4	Host Density	1	137.2880	3497.56	<0.0001
	Diet	1	0.0003	0.01	0.9295
	Block	4	0.1092	2.78	0.0758
	Density×Diet	1	0.1445	3.68	0.0791
	Error	12	0.0393		

Host density was either high or low, and diet consisted of either *E. coli* alone or *E. coli* and *S. typhimurium* mixture.

**Figure 3:**
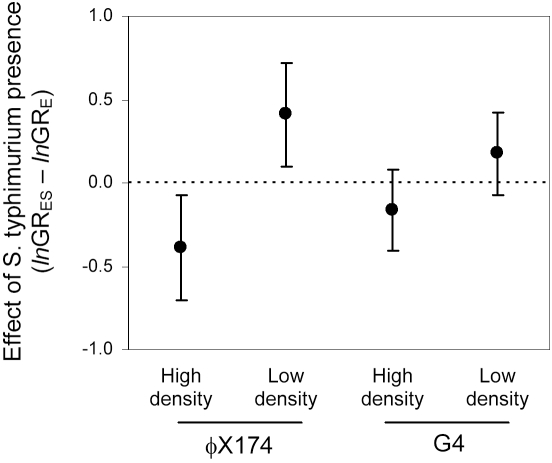
The generalist phenotype of φX174 is costly at high host density (10^8^ bacteria/mL) but advantageous at low host density (10^6^ bacteria/mL). Data are the differences between *ln*(growth rate) in mixed host cultures containing *E. coli* and *S. typhimurium* (ES) and *ln*(growth rate) in pure host cultures containing only *E. coli* (E). The dotted line represents equal growth in both host composition treatments. Each data point represents the mean of 5 replicates±95% confidence intervals.

In figure 3, we illustrated the nature of this interaction effect by plotting the differences between *ln*(growth rate) in mixed host cultures containing *E. coli* and *S. typhimurium* and *ln*(growth rate) in pure host cultures containing only *E. coli*. For the generalist phage φX174, the presence of *S. typhimurium* (the less profitable host) in experimental microcosms significantly decreased growth rate at high host density, but significantly increased growth rate at low density. In contrast, host composition had no significant effect on the growth rate of G4 at either host density.

### Evolutionary dynamics

To determine whether adaptation to low host density – but not high host density – is accomplished by the evolution of generalist traits, we adapted replicate phage populations to experimental low density and high density microcosms containing an equal mixture of *E. coli* and *S. typhimurium*. Phage populations were subjected to chemical mutagenesis followed by 10 serial transfers (10–20 generations) into either low or high host density cultures.

**Figure 4:**
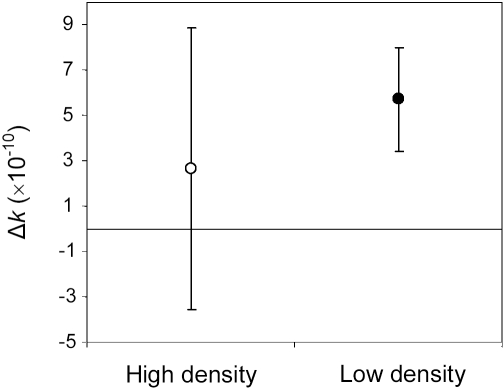
Evolutionary response in the attachment rate to *S. typhimurium* during adaptation to two-host microcosms. For individual populations, responses were calculated as *k*_10_−*k*_0,_ where *k*_i_ was the mean attachment rate measured after *i* generations (passages) of adaptation to growth at high or low host density. Data are grand means±95% confidence intervals based on four replicate evolution experiments. The black dot (low density) indicates a difference significantly greater than 0 (1-tailed paired *t*-test, *P* = 0.0056).

Low host density was expected to impose selection to incorporate the less profitable host *S. typhimurium* into the diet, and to result in an increased rate of attachment to *S. typhimurium*. In contrast, high host density was expected to impose selection to specialize on the more profitable host *E. coli*, and to result in a reduced rate of attachment to *S. typhimurium*. To determine if attachment rate was, in fact, a target of selection in our experiments, we measured the attachment rates to *S. typhimurium* for each mutagenized population both before and after evolution (fig. 4). As expected, the mean attachment rate of populations adapted to the low host density treatment was significantly higher than the mean of the ancestral populations (*t* = 5.6076, *P* = 0.0056), whereas the mean attachment rates of populations adapted to the high density treatment did not change significantly over the course of the experiment (*t* = 0.9633, *P* = 0.2032).

We had hoped to perform identical experiments using G4 populations, but were unable to generate any mutant phage capable of growth on *S. typhimurium*. We screened 1.46×10^9^ un-mutagenized phages and 2×10^4^ mutagenized phages for the ability to infect *S. typhimurium*, but found no such mutants. 2×10^4^ mutagenized phages is 20 times greater than the number of phages used to initiate individual φX174 evolution experiments, allowing us to conclude that genetic constraints prevent the evolution of host generalization in G4 populations.

### Optimal foraging in natural populations

We also investigated whether conclusions drawn from observations in φX174 (i.e. specialists favored when *N* = 10^8^; generalists favored when *N* = 10^6^) can be generalized to other phages with other life history strategies. In particular, we wanted to determine whether the life history strategies observed in marine phages, and the host densities observed in marine environments are predicted to favor specialist or generalist phages.

**Figure 5:**
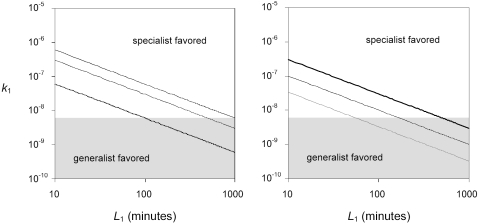
Optimal diet breadth given observed life history strategies in phages. Lines depict the attachment rate and lysis time combinations at which generalist and specialist phages should achieve equal growth rates for particular host combinations and densities, as predicted by equation 7 (*k*_1_ = *x*/[(1*−x*)*L*_1_ *N*]). Here, we assume that the second best host is a proportion *x* as profitable as the best host, and specify a particular host density *N*. *k*_1_ and *L*_1_ represent the attachment rate and lysis time, respectively, of the most profitable host. Combinations above each line favor specialists; combinations below each line favor generalists. The shaded portion of the graph indicates the region over which phage life history traits have been observed. (A) Observed attachment rate and lysis times overlap the region that favors specialists only when host density is high. Here we fixed *x* = 0.75, and examined the cases where *N* = 5×10^5^ (solid line), 1×10^6^ (dashed line), and 5×10^6^ hosts/ml (dotted line). (B) Observed attachment rate and lysis times overlap the region that favors specialists only when differences in host profitability are large. Here we fixed *N* = 1×10^6^, and examined the cases where the second best host is 75% (solid line), 50% (dashed line), and 25% (dotted line) as profitable as the best host.

We asked the following question. Given that a phage already consumes the most profitable host (host 1) with profitability *Pr*_1_ = ln*B*_1_/*L*_1_, and inhabits an environment with host density *N*, should the phage consume host 2 that is a fraction *x* as profitable (i.e. *Pr*_2_ = *x Pr*_1_)? By substituting *xPr*_1_ for *Pr*_2_ in equation 5, and rearranging the resulting inequality, we find that phage should consume host 2 only if the following inequality is met:
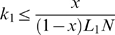
(7)We used equation (7) to identify the attachment rate and lysis time combinations that favor specialists when host densities are close to the maximum (5×10^6^ hosts/ml), mean (1×10^6^), and median (5×10^5^) observations from natural marine environments [14], [26] (Figure 5A), and when the second most profitable host in the environment is 75%, 50%, or 25% as profitable as the most profitable host (Figure 5B).

Among marine phages, observed attachment rate constants fall between 4×10^−11^ and 6×10^−9^ mL/min [20], [27]–[31] and observed lysis times fall between 40 minutes and 12 hours [30], [32]–[37] – describing a region of parameter space generally predicted to favors generalists (Figure 5). Although phages with the highest attachment rates and lysis times are predicted to specialize, the prediction only holds in rare environments where hosts are close to their maximum observed density (i.e. *N*∼5×10^6^) or in which the second best host is much less profitable than the best host (e.g., *Pr*_2_<0.5 *Pr*_1_).

## Discussion

Our results confirm that optimal foraging models of diet composition developed to describe the ecological consequences of predator foraging decisions accurately describe the ecological dynamics of bacteriophage populations in laboratory microcosms. Reproductive rates of the generalist and specialist bacteriophages φX174 and G4 conformed to the theoretical prediction that the reproductive advantage associated with host specialization depends on host density. The generalist phenotype of φX174 was advantageous at low host density (10^6^ hosts/ml), but costly at high host density (10^8^ hosts/ml).

The qualitative similarity between these results and observations from marine environments – that phages collected from higher host density coastal waters have a greater tendency toward host specialization than phages collected from lower host density pelagic ocean waters [12]–[14] – suggests that diet breadth models may also explain bacteriophage host ranges in natural populations. However, the quantitative match between our data and observations from marine environments is not as good. In particular, the average marine environment achieves a bacterial density of approximately 10^6^/ml. As this bacterial density represents then entire bacterial community, the density of particular bacterial species is likely to be much lower. Our analysis suggests that this density is not sufficient to favor the evolution of specialist phages, and yet most phages isolated from nature infect only one or a few bacterial hosts [38].

Although optimal foraging does not appear to explain the prevalence of specialist phages in marine environments, the explanation may still be primarily ecological. For example, the scarcity of hosts (relative to phages) in the marine environment may impose intense resource competition, and host specialization may evolve as a mechanism to reduce this competition. On the other hand, we provide evidence that genetic constraints also contribute to the prevalence of specialist phages in nature.

### Optimal foraging when diet breadth is determined genetically

A second explanation for the failure of the diet width model to explain the foraging patterns of bacteriophages in nature is that bacteriophages are parasites and not predators. Unlike predators that can change diet width behaviorally, bacteriophages change diet width only through genetic mutations. Thus, bacteriophages may be constrained in their diet choice in a way that predators are not. Our data support this expectation. Although the diet width model accurately predicted the *direction* of selection, it had only a limited ability to predict the *response* to selection. The ability to predict the response to selection depended on the mutational accessibility of appropriate genotypes. φX174 populations that were capable of generating appropriate genetic variation adapted to low host density as predicted, by increasing utilization of the less profitable host. In contrast, G4 populations were unable to produce mutations that allowed infection of *S. typhimurium* and were, therefore, unable to evolve a generalist phenotype.

We considered whether the inability of G4 populations to evolve a generalist phenotype resulted because G4 has less evolutionary potential than other phages, but we think this possibility unlikely. In particular, the fact that the φX174 genome encodes an identical set of genes, but possesses a generalist phenotype, indicates that these phages are not characterized by unusual functional constraints that prevent a generalist phenotype. Although phages that use alternative mechanisms of attachment (e.g. tailed phages which attach to their hosts through an appendage instead of directly with their capsid components) may be less evolutionarily constrained than G4, it is also possible that specialist phages often occupy regions of sequence space from which generalist genotypes are inaccessible.

Genetic constraints that act to limit host range in environments where hosts are rare would seem to pose a huge problem for bacteriophage survival and reproduction. One way that bacteriophage may get around this problem is to use a vertical mode of transmission (lysogeny) when host density is too low for horizontal transmission (lysis) to be effective [39]. Lysogeny is a process by which bacteriophage genomes integrate into the genome of the host, and are transmitted to the progeny during replication of the host genome. Some phages are also capable of entering into a pseudolysogeny state in which the viruses stay in a limited lytic mode, but still allow the host bacteria to grow and divide, ensuring their vertical transmission [40]. Both lysogeny and pseudolysogeny occur in marine environments [41], and moreover, there is evidence that the proportion of bacteria containing lysogenic phage is negatively correlated with bacterial density [42], [43]. This correlation is consistent with the optimal foraging predictions made here. The disadvantage of host specialization, and the corresponding advantage of lysogeny, is predicted to decrease with increasing bacterial density.

We conclude with the final caveat that attempts to explain the evolution of bacteriophage diet width using purely ecological models may be limited by the extent to which the underlying parameters search time, handling time, and energy content can themselves evolve. The difficulty arises from the difference between bacteriophage parasites and predators discussed above – bacteriophage diets are determined genetically and approach optimality over an evolutionary rather than ecological timescale. The limited utility of the diet width model for predicting phenotypic evolution was apparent in the evolution experiments in which we adapted phage populations to a high host density. The diet width model and our ecological experiments both indicated that high host density would impose selection for reduced consumption of the inferior host *S. typhimurium*. Consumption of *S. typhimurium* is mediated by the rate of attachment to *S. typhimurium*, but this trait did not evolve in the high host density evolution experiments. A possible explanation for this outcome is that selection was also acting on lysis time and burst size [10], [44], [45] to increase the profitability of *S. typhimurium*. If host profitabilities and diet width evolve simultaneously, optimal diet choice becomes a chicken and egg problem. In cases where parasite diets do appear optimal, two explanations are possible. Parasite diet breadth could have evolved to match available host profitabilities, or host profitabilities could have evolved to match the current diet.
